# The Role of Myocardial Mitochondrial Quality Control in Heart Failure

**DOI:** 10.3389/fphar.2019.01404

**Published:** 2019-12-06

**Authors:** Zhiling Qiu, Yi Wei, Qingqiao Song, Bai Du, Huan Wang, Yuguang Chu, Yuanhui Hu

**Affiliations:** ^1^Department of General Diseases, Guang'anmen Hospital, China Academy of Chinese Medical Sciences, Beijing, China; ^2^Department of Cardiology, Guang'anmen Hospital, China Academy of Chinese Medical Sciences, Beijing, China

**Keywords:** heart failure, mitochondrial quality control, mitochondrial biogenesis, mitochondrial dynamics, mitophagy

## Abstract

At present, the treatment of heart failure has entered the plateau phase, and it is necessary to thoroughly study the pathogenesis of heart failure and find out the corresponding treatment methods. Myocardial mitochondria is the main site of cardiac energy metabolism, whose dysfunction is an important factor leading to cardiac dysfunction and heart failure. Mitochondria are highly dynamic organelles. Continuous biogenesis, fusion, fission and mitophagy, contribute to the balance of mitochondria's morphology, quantity, and quality, which is called mitochondrial quality control. Mitochondrial quality control is the cornerstone of normal mitochondrial function and is found to play an important role in the pathological process of heart failure. Here, we provide an overview of the mechanisms of mitochondrial quality control and recent studies on mitochondrial quality control in heart failure, hoping to provide new ideas for drug development in heart failure.

## Introduction

Heart failure has become a severe health care problem, especially in elderly population, all over the world. It is the end stage of various cardiovascular diseases, characterized by high morbidity, high hospitalization rate and high mortality (Benjamin et al., 2017). Current research suggests that the basis of heart failure is myocardial energy metabolism remodeling and ventricular remodeling. Cardiomyocytes are highly metabolically active with mitochondria as their major energy source, which account for about one-third of the volume of cardiomyocytes (Hall et al., 2014). In addition to energy production, mitochondria play central role in a variety of intracellular events, such as calcium signaling, apoptosis, and electrolyte homeostasis. Moreover, the mitochondrial respiratory chain is the main site of oxygen free radical production, and has the dual identity of the source and target of cellular oxidative stress damage. Therefore, mitochondria have been considered as important targets for the development of new therapeutic interventions for cardiovascular diseases, especially heart failure. Recent studies have shown that mitochondrial quality control (MQC) is the basis for maintaining the stability and integrity of mitochondrial structure and function, and is an important defense mechanism for cells to survive from mitochondrial damage (Hurtley, 2015). This review article is to summarize the current progress of research in MQC mechanisms and their functions in heart failure.

## Mechanism of MQC

**Figure 1:**
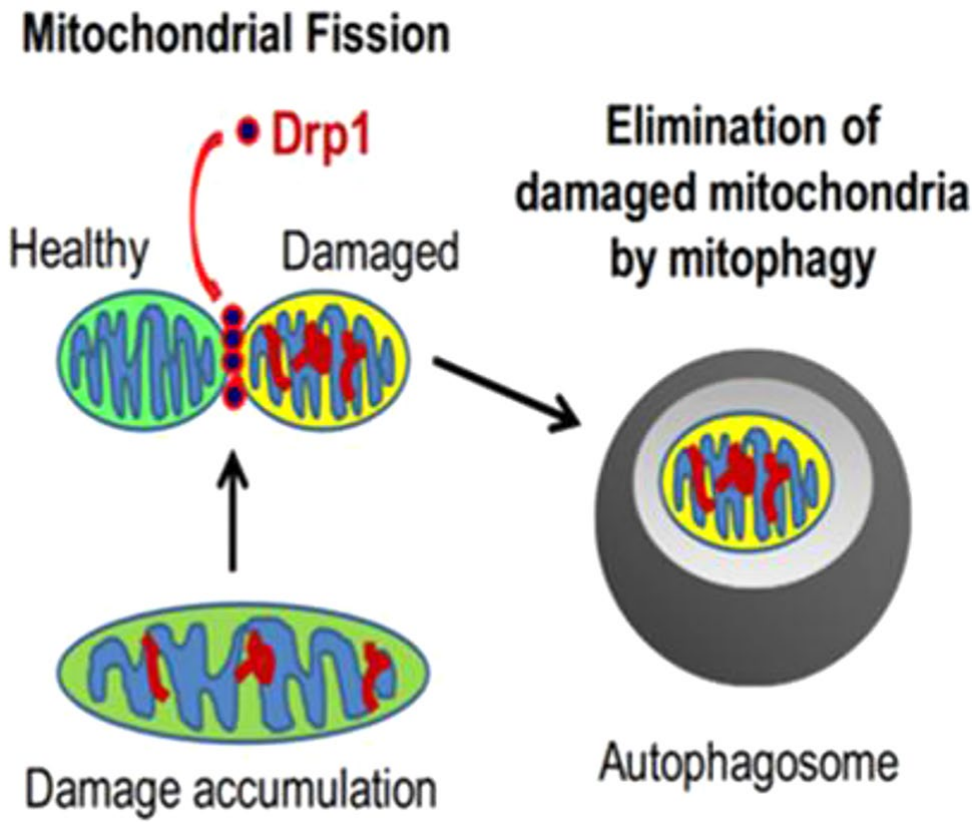
The relationship between mitochondrial fission and mitophagy (Ikeda et al., 2015).

Mitochondria are highly dynamic organelles that maintain a relatively stable quantity and quality through constant fusion, fission, biogenesis and mitophagy, which is the essential components of MQC. Mitochondria form a network of tubes through the fusion of inner and outer membranes for information exchange and mitochondrial DNA (mtDNA) damage repair (Archer, 2013); unrepairable damaged mtDNA will be separated into mitochondria with low membrane potential during fission, which later will be recognized by mitophagy-related proteins and selectively cleared by the mitophagy pathway (**Figure 1**) (Twig et al., 2008; Youle and Narendra, 2011); in addition, the process, consisted of mtDNA replication regulated by genome within the nucleus and synthesis of mitochondrial precursor proteins, is called mitochondrial biogenesis (Scarpulla, 2008), which is followed by division into two daughter mitochondria in response to cell proliferation, increased energy demand, and expansion of the number of mitochondria when mitochondrial damage is severe. At present, the research on mitochondrial quality control is increasing, and its specific mechanisms are divided into the following aspects:

### Mitochondrial Biogenesis

Mitochondria are organelles with relatively independent genetic systems and biosynthetic sites. Most of the proteins assembled in mitochondria are encoded by nuclear DNA (nDNA), which are translocated into mitochondria after translation. mtDNA encodes only a small number of proteins, and the transcription and translation of these proteins are regulated by proteins encoded by nDNA (Scarpulla, 2002). The newly synthesized DNA, RNA and proteins enhance the ability of mitochondrial oxidative phosphorylation and ATP synthesis, and expand the contents of mitochondria, which is closely related to the regulation of mitochondrial function. Defective mitochondrial biogenesis will result in decreased mitochondrial number and ATP production, leading to mitochondrial dysfunction.

Current studies suggest that peroxisome proliferator-activated receptor gamma coactivator 1α (PGC-1α) is the most important regulator of mitochondrial biogenesis (Scarpulla et al., 2012; Wenz, 2013). PGC-1α has specific tissue distribution, and mainly expresses in tissues with high energy requirements or high oxidative activity, such as heart, skeletal muscle, kidney, liver, etc., which suggests that it is closely related with energy metabolism of the body. PGC-1α is a transcriptional cofactor rapidly induced under conditions of high energy consumption, such as cold, exercise, and fasting (Senoo et al., 2015; Kong et al., 2014). Although PGC-1α cannot directly bind to mtDNA, it binds and strongly enhances the activity of respiratory factor 1/2 (NRF-1/2), which promotes mtDNA replication, transcription and protein formation, and subsequently promotes cellular respiration (Kang and Li, 2012). The replication and transcription of mtDNA are mainly regulated by mitochondrial transcription factor A (Tfam). The promoter of Tfam contains a binding site for NRF-1 or NRF-2 (Dong et al., 2002), allowing mitochondrial biogenesis to achieve co-regulation between mitochondria and nuclear activation *via* the PGC-1α-NRF-1/2-Tfam pathway.

In addition, SIRT1 and SIRT3 in the highly conserved type III histone deacetylase family (Sirtuins, SIRTs) are other factors closely related to the regulation of mitochondrial biogenesis. SIRT1 is a sensitive energy sensor in mammalian myocardial tissue, mainly expressed in the cytoplasm, and translocates to the nucleus under stress (Sundaresan et al., 2011). SIRT3 is mainly expressed in mitochondria, which is identical to SIRT4 and SIRT5 in the Sirtuins family (Houtkooper et al., 2012). However, studies have shown that mitochondrial proteins in SIRT3 knockout mice are at high acetylation levels, whereas those in SIRT4 and SIRT5 knockout mice are not, indicating that SIRT3 is the major deacetylase in mitochondria (Lombard et al., 2007). Although SIRT1 and SIRT3 are located at different locations in the cell, they have a synergistic effect on mitochondrial biogenesis, which was called the SIRT1/SIRT3 dual regulatory axis by Brenmoehl and Hoeflich, (2013). Studies have shown that SIRT1 is an upstream regulator of PGC-1α, and overexpression of SIRT1 can enhance the deacetylation of PGC-1α and promote mitochondrial biogenesis (Khan et al., 2015). PGC-1α promotes the binding of the transcription factor estrogen-related receptor α (ERRα) to the ERRα response element located in the SIRT3 promoter, which in turn activates the expression of SIRT3 (Kong et al., 2010).

### Mitochondrial Dynamics

In 1914, Lewis and Lewis (1914) first proposed that mitochondria change their shape by frequent fusion and fission, thereby maintaining the stability of their network structure, which was called mitochondrial dynamics. Mitochondria sometimes split into short rods and ellipse, and sometimes merge into a linear or network shape. In most situations, mitochondria fuse and extend into a tubular network structure, which is beneficial for close contact with other organelles (such as the endoplasmic reticulum), and also facilitates the sharing of components between mitochondria, exchanges mitochondrial contents, repairs damage, and separates the parts that cannot be repaired. This shifting dynamic allows mitochondria to adapt to different physiological needs of cell proliferation, differentiation and environmental changes.

Mitochondria fusion and fission, mainly happen in the inner and outer membranes of mitochondria, are controlled by a group of dynamin-related regulatory proteins containing a conserved GTPase domain (Scott and Youle, 2010). Mitochondria fission only occurs in the mitochondrial outer membrane, and its regulatory proteins include dynamin-related protein 1(Drp1), mitochondrial ﬁssion protein 1(Fis1), and mitochondrial fission factor (MFF). Drp1 mainly locates at the fission compression site of cytoplasm and mitochondria. Since it does not have transmembrane structure, a receptor is needed for binding to the outer membrane of mitochondria. The Drp1 receptor proteins include Fis1, MFF and recently identified mitochondrial dynamics protein of 49 and 51 kDa (Mid49/51) (Losón et al., 2013; Otera et al., 2010). Fis1 has multiple transmembrane helical structures, and mainly colocalizes with MFF and Mid49/51 in mitochondrial outer membrane. During fission, they recruit and bind to cytoplasmic Drp1, and distribute in a point-like manner at the potential fission-compression site of the mitochondrial outer membrane, forming a circular structure and initiating subsequent mitochondrial fission (Suliman and Piantadosi, 2016; Elgass et al., 2013).

Mitochondrial fusion is mainly composed of mitochondrial inner membrane fusion and outer membrane fusion. Mitochondrial fusion protein 1 (mitofusion 1, Mfn1) and mitochondrial fusion protein 2 (mitofusion 2, Mfn2) are the major regulatory proteins of mitochondrial outer membrane fusion. In mitochondrial fusion, Mfn1 or Mfn2 in two adjacent mitochondrial outer membranes form a dimeric or heterodimeric structure, causing fusion of the mitochondrial outer membrane (Khan et al., 2015). It is important to note that mitochondrial fusion will be functional unless both Mfn1 and Mfn2 are knocked out, which will eventually lead to fragmentation of mitochondria (Formosa and Ryan, 2016). Optic atrophy 1 (Opa1) is a major regulatory protein of mitochondrial inner membrane fusion, locating in the intermembrane space of mitochondria. Its main function is to maintain the stability of mitochondrial cristae, to remodel the mitochondrial inner membrane and to maintain the integrity of the respiratory chain. During the fusion process, inactive Opa1 is degraded into long and short forms, and the long version is important for initiating mitochondrial inner membrane fusion (Griparic et al., 2007).

### Mitophagy

**Figure 2:**
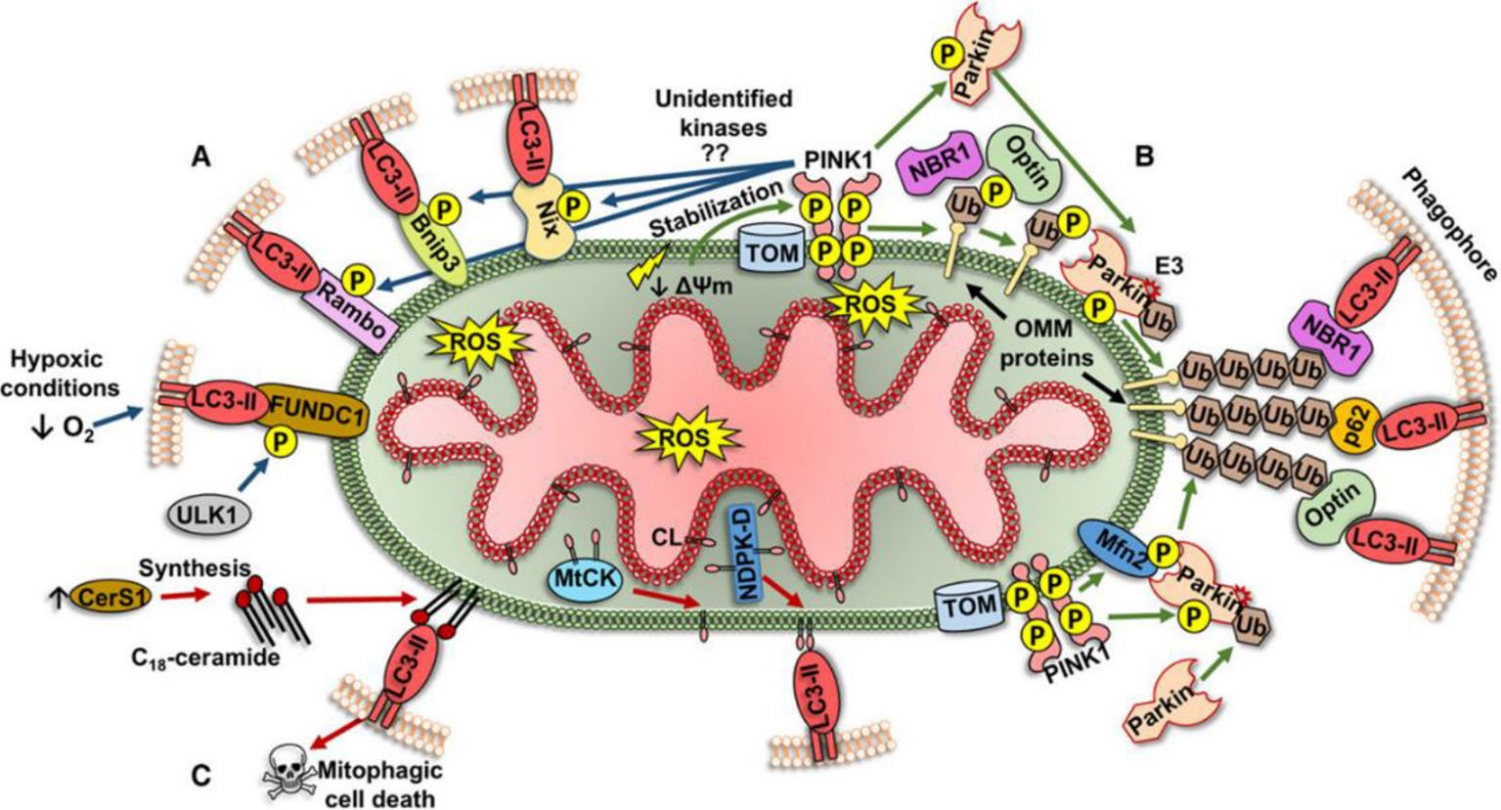
Three different types of mitophagy pathway (Ploumi et al., 2017).

Initially, autophagy was thought to be a non-selective degrading of cytoplasmic proteins and organelles to achieve cell self-renewal and maintain metabolism. However, current studies have shown that autophagy can also selectively degrade protein aggregates (Tannous et al., 2008) as well as organelles such as endoplasmic reticulum (Bernales et al., 2007) and mitochondria. In 2004, Ingrid Kissova et al. (2004) first discovered the phenomenon of selective degradation of mitochondria in yeast. In 2005, Lemasters et al. (2005) started to use the term "mitophagy". Mitophagy is closely related to mitochondrial fission. Damaged mitochondria produce more than ten times reactive oxygen species (ROS) than healthy mitochondria. Large amount of ROS can directly damage mitochondrial proteins and DNA, aggravating mitochondrial dysfunction. Unrepairable damaged mitochondrial protein and DNA will be packed into a new mitochondrion through fission, and eliminated by mitophagy, which plays an essential role in maintaining mitochondrial and cellular functions. Currently, mechanisms behind mitophagy can be classified into three different types as following (**Figure 2**) (Ploumi et al., 2017):

1. Mitochondrial outer membrane receptor-mediated mechanism. The proteins constituting and localizing to the outer membrane of mitochondria (Bnip3, Nix, FUNDC1, and Bcl-Rambo) can directly regulate the phosphorylation of LIR domain, and bind to lipidated LC3 (LC3-II) to form autophagosomes.
2. The mechanism of the pink1/parkin pathway. Changes in mitochondrial membrane potential (DΨm) lead to the activation of pink1, followed by the target proteins of pink1 (Ubiquitin/Ub and Mfn2) recruit E3 ligase parkin to the mitochondrial outer membrane. Under the pink1-dependent activation, parkin can ubiquitinate many proteins in the mitochondrial outer membrane. These ubiquitinated proteins recruit specific autophagy-related receptors (p62/SQSTM1, NBR1, and optineurin/optin) binding to LC3-II to form autophagosomes.
3. Lipid receptor-mediated mechanisms. Lipids locating in the outer membrane of mitochondria can also act as receptors for mitophagy. In damaged mitochondria, cardiolipin (CL) is transferred to the mitochondrial outer membrane *via* a specific transporter (MtCK, NDPK-D) and directly binds to LC3-II on phagocytes for eliminating damaged mitochondria.

## Current Research Progress on Myocardial Mitochondrial Quality Control in Heart Failure

The highly precise coordination of myocardial mitochondrial biogenesis, fusion, fission, and mitophagy determines the morphology, quantity, and quality of mitochondria and directly affects mitochondrial function. Defective mitochondrial quality control leads to mitochondrial dysfunction, which may induce dysfunction or even death of cardiomyocytes, eventually leading to heart failure. In recent years, the quality control of myocardial mitochondria in heart failure has gradually gained attention, and there is a complex interactive network among mitochondrial biogenesis, fusion, fission and mitophagy. The pharmacological intervention targeting on this network may create a new treatment for heart failure.

The mechanism of occurrence and development of heart failure is complex and multifaceted and has not been fully understood. Studies have shown that myocardial mitochondrial dysfunction occurs at very early stage during the pathophysiological development of heart failure. Therefore, timely recovery from mitochondrial damage has important cardiovascular protective effect (Faerber et al., 2011). In a clinical trial of heart failure, microarray analysis using myocardial tissues of patients with cardiomyopathy heart failure and healthy people revealed that the expression level of PGC-1α in myocardium of patients with cardiomyopathy heart failure was significantly lower than that of healthy people (Sihag et al., 2009). In myocardium in the late stage of heart failure, mitochondrial content and mtDNA replication were significantly reduced, and PGC-1α expression was significantly down-regulated in different types of animal heart failure models (Sun et al., 2007; Watson et al., 2007). Studies have suggested that overexpression of PGC-1α may be beneficial for the heart under pressure load (Lehman et al., 2000). However, the role of PGC-1α in heart failure is still controversial. Overexpression of PGC-1α may not improve mitochondrial function and myocardial contractile function (Pereira et al., 2014), and some studies have shown that, in the myocardial tissues of patients with congestive heart failure, there was no significant change in gene expression of PGC-1α (Hu et al., 2011). In addition, in transgenic mouse model, overexpression of PGC-1α specific in the myocardium after adulthood leads to excessive proliferation of mitochondria, structural disorder of the sarcoplasmic reticulum of cardiomyocytes, decreased myocardial contractile function, cardiac enlargement and cardiac insufficiency (Russell et al., 2004). These data suggest that PGC-1α promotes mitochondrial biogenesis, but the outcome of overexpression of PGC-1α and its effect on heart failure are closely related with the degree of overexpression and the interactions between mitochondrial biogenesis and other intracellular events. The role of PGC-1α in different types of heart failure remains to be further studied. As a sensitive energy regulator, SIRT1 translocates to the nucleus during heart failure, participates in mitochondrial biogenesis by deacetylating PGC-1α, and thereby regulates energy metabolism. Translocation of SRIT1 have been shown in different heart failure models, including TO-2 hamsters with heart failure and rats after myocardial infarction, etc. (Tanno, 2010). In a study performed by Alcendor's group (Alcendor et al., 2004), SIRT1 protected cardiomyocytes from death under stress conditions, promoted cardiomyocyte growth, and led to cardiac hypertrophy. Similarly, some studies suggest that SIRT1 expression levels are elevated in cardiac hypertrophy and heart failure (Li et al., 2009; Vahtola et al., 2008). However, similar as PGC-1α, the function of SIRT1 overexpression in heart failure is closely related to its levels (Alcendor et al., 2007). Low to moderate level of SIRT1 expression (2.5 to 7.5 times higher than endogenous level) has a protective effect on age-dependent myocardial hypertrophy, apoptosis, and cardiac insufficiency, whereas high levels (12.5-times) can induce dilation, hypertrophy, and heart failure. Similarly, in a mouse model of myocardial infarction, low levels of overexpression of SIRT1 can reduce infarct size and improve cardiac function (Hsu et al., 2010). These studies show that, like PGC-1α, the expression level of SIRT1 needs to be tightly controlled to achieve the desired effect.

A growing body of evidence suggests that impaired SIRT3 activity plays a role in many heart disorders, including heart failure. In rodent heart failure model (Grillon et al., 2012), the expression of SIRT3 decreased with increased myocardial mitochondrial protein lysine acetylation in the heart, suggesting that SIRT3 activity was impaired. The decrease in SIRT3 expression may be resulted from downregulation of PGC-1α. SIRT3 regulates many aspects of mitochondrial function by deacetylating ATP production-related enzymes in mitochondria (Palacios et al., 2009) and participating in the elimination of reactive oxygen species (Sundaresan and Gupta, 2009). Knockout of SIRT3 can cause significant myocardial mitochondrial dysfunction in energy metabolism and abnormal systolic contraction in the heart (Koentges et al., 2015). In contrast, upregulation of SIRT3 increases deacetylation of mitochondrial proteins and stabilizes mitochondrial function (Hirschey et al., 2011). The importance of SIRT3 is also demonstrated during the development of heart failure. In the early stage of heart failure, the expression of SIRT1/SIRT3 in the myocardium may be stimulated by chemical and mechanical factors to compensate the functional lost. However, when the function of the heart is seriously impaired, SIRT1/SIRT3 is down-regulated. Therefore, development of drugs targeting at up-regulation of SIRT1/SIRT3 may be an effective direction for the treatment of heart failure.

The morphology of the mitochondria (tubular or fragmented) is dependent on the activities of fusion and fission proteins, which play a crucial role in cardiac development, Ca^2+^ signaling, mitochondrial quality control, and cell death in adult cardiomyocytes. Damage to this complex circuit can lead to dysfunction and death of cardiomyocytes (Marín-García and Akhmedov, 2016). The dynamic changes of mitochondrial spatial morphology are closely related to mitochondrial metabolism and oxidative phosphorylation. Studies have found that hypoxia can cause large changes in mitochondrial structure and dynamics, ultimately leading to mitochondrial dysfunction, decreased ATP supply, and activation of cell death pathways (Solaini et al., 2010; Khacho et al., 2011). After conditionally knocking out Mfn1 and Mfn2 genes in adult rats, the mitochondrial fission in cardiomyocytes increased obviously, leading to abnormal cellular respiration, eventually leading to progressive dilated cardiomyopathy (Chen et al., 2011). Chen L et al. observed that mitochondria in the adult SD (Sprague-Dawley) rat model of heart failure after myocardial infarction were massively fragmented. Although the mRNA level of Opal did not alter, the protein content of Opal was significantly decreased, while the protein contents of Mfnl and Mfn2 did not change. Therefore, the decrease of protein content of Opa1 may be related to post-translational modification of protein (Chen et al., 2009). Other studies have shown (Sabbah et al., 2014) that in the heart failure dog model, mitochondrial fission and fusion proteins in left ventricular myocardium are dysregulated. Compared with the normal canine myocardial tissue, the expression levels of the fusion protein Mfn2 and Opal in the heart failure model were significantly down-regulated, but the expression levels of the fission proteins Drpl and Fisl were significantly up-regulated. A study showed that Mff mutant mice died at 13 weeks due to heart failure caused by severe dilated cardiomyopathy. Mutant tissues showed decreased mitochondrial density and respiratory chain activity, and increased mitochondria. It means that Mff-mediated mitochondrial division promotes the development of heart failure (Chen et al., 2015). Similarly, a study confirmed that homozygous Mff-deficient (Mffgt) mice exhibited smaller infarct size, restored cardiac function, improved blood flow, and reduced microcirculatory perfusion defects (Zhou et al., 2017).

In heart failure, myocardial mitochondrial fusion–fission becomes imbalanced, favoring mitochondrial fission, which leads to declining mitochondrial function, mitochondrial autophagy, and ultimately cardiomyocyte death. Normally, mitophagy has a protective effect on cardiomyocytes, which is important for the clearance of dysfunctional mitochondria, cell renewal, and cell survival, especially for terminally differentiated cardiomyocytes with limited regenerative ability (Moyzis et al., 2015). On the other hand, insufficient or excessive mitophagy aggravates the development of heart failure. Current research on mitophagy in cardiovascular diseases is mainly focused on the pink1/parkin pathway. Growing evidence suggests that different interventions in the pink1/parkin pathway-mediated mitochondrial regulation can effectively prevent heart failure (Shires et al., 2018; Mukherjee et al., 2015).In a recent study, adenosine monophosphate activated protein kinase (AMPK) in myocardial tissue samples from heart failure patients and transverse aortic coarctation (TAC)-induced mouse heart failure models was found to accelerate isoform switching from AMPKα2 to AMPKα1, accompanied by a decrease in mitophagy and mitochondrial function (Wang et al., 2018). Overexpression of AMPKα2 can enhance the signaling of pink/parkin pathway by phosphorylation, enhance mitophagy, promote the elimination of damaged mitochondria, improve mitochondrial function, reduce ROS production and cardiomyocyte apoptosis, and thereby prevent the early progress of heart failure. Pink1or parkin knockout mice have impaired mitochondrial function, increased myocardial infarct size, systolic dysfunction and higher sensitivity to myocardial ischemic injury, while overexpression of pink1 and parkin can reduce the damage of cardiomyocytes (Rüb et al., 2017; Kubli et al., 2013). There are also some studies on mitophagy mediated by other pathways. One study has shown that inhibition of FUNDC1-dependent mitophagy disrupts mitochondrial homeostasis and promotes the development of cardiac ischemia-reperfusion injury (Zhou et al., 2018). DUSP1 and its downstream JNK pathway are therapeutic targets for conferring protection against IR injury by repressing Mff-mediated mitochondrial fission and Bnip3-required mitophagy (Jin et al., 2018). Sustained overload stress in the model of heart failure induced by ligation of ascending aorta leads to excessive mitophagy. Inhibitors of mitochondrial fission can effectively inhibit mitophagy, reduce myocardial fibrosis, and thereby improve cardiac function (Givvimani et al., 2012). Therefore, the timing for properly inducing or inhibiting myocardial mitophagy will be critical for potential treatments of heart failure targeting at mitophagy.

## Conclusion

Mitochondrial dysfunction plays an important role in the pathophysiological process of heart failure development. Mitochondria are not only the main site of energy metabolism, but also involved in regulating cell metabolism, signal transduction, apoptosis, etc., which determine the survival of cells. Appropriate mitochondrial quality control is an important defense mechanism for cardiomyocytes to cope with various stress or injuries. The results from current research show that the regulation of mitochondrial quality control can effectively enhance the synthesis of ATP in cardiomyocytes, maintain the quality and quantity of mitochondria, regulate myocardial energy metabolism, inhibit the apoptosis of cardiomyocytes, inhibit the pathological remodeling of myocardial cells from metabolism to structure, reduce myocardial ischemic injury, and thereby delay the pathological progress of heart failure.

At present, research on mitochondrial quality control in heart failure is still in its early phase, and some research results are controversial. The reasons accounting for this situation include: 1) Mitochondrial biogenesis, mitochondrial dynamics, and mitophagy are all involved in the development of heart failure, and this extreme dynamic system makes it very difficult to systemically study the functions of MQC in heart failure; 2) MQC plays different roles during the progress of heart failure and is affected by various pathological conditions. Therefore, further studies are needed to elucidate the mechanisms behind the functions of MQC in heart failure and provide new insights, such as precise timing and new target molecules, for the development of innovative therapeutics for heart failure.

## Author Contributions

YH and BD conceived the topic. HW and YW helped to draft the paper. YC and QS consulted the references. ZQ wrote the paper.

## Funding

This research was supported by National Natural Science Foundation of China (81673971).

## Conflict of Interest

The authors declare that the research was conducted in the absence of any commercial or financial relationships that could be construed as a potential conflict of interest.
